# Nomogram incorporating Epstein-Barr virus DNA and a novel immune-nutritional marker for survival prediction in nasopharyngeal carcinoma

**DOI:** 10.1186/s12885-023-11691-8

**Published:** 2023-12-09

**Authors:** Shuting Wu, Xiaofei Yuan, Haoran Huang, Yanfei Li, Linchong Cui, Danfan Lin, Wenxuan Lu, Huiru Feng, Zilu Chen, Xiong Liu, Jiajie Tan, Fan Wang

**Affiliations:** Department of Otolaryngology-Head and Neck Surgery, Nanfang Hospital, Southern Medical University, Jingxi Street, Baiyun District, Guangzhou, 510515 P.R. China

**Keywords:** Nasopharyngeal carcinoma, Lymphocytes × albumin, Epstein–Barr virus DNA, Nomogram, Survival

## Abstract

**Background:**

Since Immune response, nutritional status and Epstein–Barr Virus (EBV) DNA status have been confirmed to be relevant to the prognosis of patients with nasopharyngeal carcinoma (NPC), we believe that the combination of these factors is of great value for improving the predictive ability. LA (lymphocytes × albumin), a novel indicator, had not been studied yet in NPC. We combined it with EBV DNA and used nomograms to increase the accuracy of prognosis.

**Methods:**

A total of 688 NPC patients were retrospectively reviewed and further divided into training and validation cohort randomly. Kaplan–Meier analyses were used to to distinguish the different survival outcomes. Multivariate Cox analyses were used to identify the independent prognostic factors for progression-free survival (PFS) and overall survival (OS). Calibration curves, concordance indexes (C-indexes) and decision curve analyses (DCA) were used to evaluate the nomograms’ predictive value.

**Results:**

Patients with low LA and positive EBV DNA correlated with poorer 5-year PFS and OS (all *P* < 0.005). In multivariate Cox analyses, LA and EBV DNA were both confirmed to be independent prognostic factors for PFS and OS (all *P* < 0.05). Prognostic nomograms incorporating LA and EBV DNA achieved ideal C-indexes of 0.69 (95% CI: 0.65–0.73) and 0.77 (95% CI: 0.71–0.82) in the prediction of PFS and OS. Otherwise, the calibration curves and DCA curves also revealed that our nomograms had pleasant predictive power.

**Conclusions:**

LA is a novel and powerful biomarker for predicting clinical outcomes in NPC. Our nomograms based on LA and EBV DNA can predict individual prognosis more accurately and effectively.

**Supplementary Information:**

The online version contains supplementary material available at 10.1186/s12885-023-11691-8.

## Introduction

Nasopharyngeal carcinoma (NPC), a distinctive head and neck cancer arising from nasopharyngeal epithelium, has a high reported incidence of 5.0/100,000 in Southeast Asia [1]. Despite survival rates have increased with the advance of multidisciplinary management and treatment, approximately 20% of patients still experience disease progression and mortality [2–4]. Therefore, to improve the treatment effects, identifying key predictors to select patients at different risk levels is required for individualized treatment.

The dynamic interaction between immune response and tumor microenvironment has become a popular focus of attention. Increasing evidence shows that the immune response of a patient contributes to cancer development and progression [5–7]. Lymphocytes, a crucial type of immune cells, play an essential role in immune monitoring by inhibiting the proliferation and metastasis of cancer cells via lymphocyte-mediated cytotoxicity [8]. And its level can reveal prognosis of cancer. Okadome [9] et al. found that the low tumor-infiltrating lymphocytes (TILs) were associated with poor overall survival (OS) in esophageal carcinoma. In addition, nutritional status is linked to therapeutic response, treatment toxicity and prognosis of various cancers [10, 11]. Serum albumin (ALB), a classical biomarker of the body’s nutritional status, can reliably assess the nutritional status of cancer patients. Decreased albumin level is a sign of poor prognosis for cancer [12–15]. Li et al. suggested serum albumin as an effective prognostic biomarker of NPC patients [16]. Using a combination of these factors of immune and nutritional status, a novel indicator, LA (Lymphocytes × albumin), caught our interest. The LA, calculated by the product of lymphocytes and albumin, was first proposed in distinguish benign and malignant pancreatic cystic neoplasm [17]. Recently, it had been demonstrated that low LA was particularly related to poor survival outcomes in rectal cancer [18]. However, the prognostic value of LA in NPC is still unclear, and using LA alone is still insufficient for making individualized predictions.

As a specific pathogenic factor of NPC, the contribution of Epstein-Barr virus (EBV) DNA cannot be ignored [19]. The detection of EBV DNA level in plasma is extensively used in population screening, prognosis evaluation and risk stratification [20–22]. In plasma samples, the sensitivity and specificity of EBV DNA in screening for nasopharyngeal carcinoma were 97.1% and 98.6%, respectively [21]. High plasma EBV DNA concentration was significantly correlated with advanced tumor stage and worse prognosis [20, 23]. Many scholars further revealed that EBV DNA, in combination with other indicators, strengthened the predictive efficacy [24–26]. For example, Jin [25] combined EBV DNA with the systemic inflammation response index (SIRI) and found that it achieved the largest area under the curve (AUC) to predict survival. And in Huang’s study, the predictive capacity of EBV DNA combined with the C-reactive protein/albumin ratio (CAR) has a higher C-index of 0.693, which was superior to EBV DNA or CAR alone [26]. Therefore, we tried to combine LA and EBV DNA to improve the predictive ability.

Nomogram, a more precise tool for individual prediction of a clinical event than traditional TNM staging system, is commonly used for risk estimation in various types of cancers [27]. In the nomogram developed by Wang et al., we can intuitively and accurately predict the survival probability for individual patients with small-cell lung cancer with a higher C-index of 0.722 [28]. Hence, in the present study, we initially attempted to show the prognostic significance of LA in NPC and then investigated the effect of its combination with EBV DNA on improving prediction. Finally, we established nomograms to further enhance the predictive precision for individual patients in NPC.

## Materials and methods

### Study population

Between January 2005 to December 2015, a retrospective study was performed including 688 patients with NPC, who were diagnosed at the Nanfang Hospital of Southern Medical University. The inclusion criteria were as follows: 1) histopathology confirmed NPC; 2) had complete medical records and baseline laboratory data including pretreatment albumin, lymphocytes and EBV DNA; 3) completed the entire treatment. The exclusion criteria were as follows: 1) developed distant metastasis or combined with other malignancies at diagnosis; 2) had a history of cancer treatment; 3) had serious complications; 4) had insufficient follow-up data. All patients were randomly divided into a training cohort (456 patients) and a validation cohort (232 patients). The 8th edition of the American Joint Committee on Cancer (AJCC) staging system was performed for the present study. The research was approved by the Ethics Committee of Nanfang Hospital of Southern Medical University (Ethical review approval no.: NFEC-2017–165).

### Data collection

The peripheral blood test of including lymphocytes, albumin (ALB) were gathered within 1 week before treatment. The plasma EBV DNA was tested within 1 month before treatment. Both peripheral blood test and EBV DNA measurement were conducted in the Laboratory Medicine Center of Nanfang Hospital, Southern Medical University from the standard operating procedures (The method of EBV DNA detection in Supplementary file 1, Additional File 1).

The BamH I-W region of EBV genome was amplified by real-time quantitative polymerase-chain reaction (RT-qPCR) technique to determine plasma EBV DNA levels. After PCR assay, the plasma EBV DNA level of ≥ 500 copies/ml was defined as positive, and the negative EBV DNA level was recorded as < 500 copies/ml. Referring to previous studies [29, 30], we chose 500 copies/ml as the optimal cutoff value based on the referring threshold of Laboratory Medicine Center, Nanfang Hospital, Southern Medical University. The calculation formula of LA was described as follows: LA = total lymphocyte count (10^9^/L) × serum albumin (g/L). The cutoff of LA was obtained via receiver operating characteristic (ROC) curve analyses.

### Treatment

Based on the National Comprehensive Cancer Network (NCCN) guidelines, intensity-modulated radiation therapy (IMRT) along was recommended for stage I patients, concurrent chemoradiotherapy (CCRT) was recommend for stage II patients, a combination of CCRT and induction chemotherapy (IC) with or without adjuvant chemotherapy (AC) was recommend for stage III/IV patients. (The detailed protocols for radiotherapy and chemotherapy were in Supplementary file 2, Additional File 2).

### Follow-up and endpoint

After the completion of treatment, patients were required to assess every 3 months for the first year, and every 6 months between the second and third year, then yearly thereafter. The examination items included physical examination, nasopharyngeal endoscopy, abdominal ultrasound, peripheral blood test, chest radiography, magnetic resonance imaging (MRI) of nasopharynx and neck, a whole-body bone scan or the positron emission tomography and computed tomography (PET-CT). The recurrence of nasopharynx and neck tumor or metastasis of cervical lymph nodes was confirmed by the biopsy or needle biopsy in the suspected region. Our primary endpoint was progression-free survival (PFS), defined as the time from diagnosis to the date of disease progression, death from any cause or last follow-up. The secondary endpoint in current study was overall survival (OS), which was defined as the time between diagnosis to death from any cause or last follow-up.

### Statistical analysis

Continuous variables were transformed into categorical variables, and chi-square test or Fisher’s exact test was performed to compare the clinical features of these two groups. The receiver operating characteristic (ROC) curve was used to determine the cut-off of LA. Survival curves were calculated using Kaplan–Meier method and compared by the log-rank test. Univariate and multivariate COX proportional hazards regression analyses were performed to determine independent prognostic factors of PFS and OS. Then, based on the significant prognostic factors from multivariate COX regression analysis in the training cohort, the OS and PFS nomogram were developed. To measure the discrimination performance of the nomogram, we calculated Concordance indexes (C-indexes). The calibration curve was used to evaluate the goodness of fit between the observed values and predicted values. Decision curve analysis (DCA) was performed to assess the clinical usefulness of the nomogram. All analyses were carried out using IBM SPSS 23.0, Graphpad Prism V6.0 and R software v4.2.1. All tests were two-tailed and a *P* value < 0.05 was considered statistically significant.

## Results

### Baseline characteristics in the training and validation cohorts

A total of 456 patients in the training cohort and 232 patients in the validation cohort were included in this study. The baseline characteristics of the training cohort and validation cohorts are shown in Table 1. There was no significant difference between the two cohorts.

**Table 1:** Baseline characteristics of the patients in training and validation cohorts

Characteristic	Training cohort (*n* = 456)	Validation cohort (*n* = 232)	*P* value
Gender (male/female)	327/129	179/53	0.126
Age (≤ 55/ > 55,years)	352/104	188/44	0.246
Smoke (no/yes)	278/178	130/102	0.213
Drink (no/yes)	415/41	202/30	0.108
WHO pathological type (I/II/III)	0/32/424	3/14/215	0.061
T stage (T1-T2/T3-T4)	179/277	104/128	0.160
N stage (N0-N1/N2-N3)	202/254	105/127	0.811
TNM stage (I-II/III-IVa)	95/361	52/180	0.633

*Abbreviations: WHO* World Health Organization, *T* Tumor, *N* Node, *TNM* Tumor node metastasis

In the training cohort, there were 327 (71.7%) male and 129 (28.3) female patients with a median age of 47 years. The median follow-up duration was 61 months. During follow-up, 128 (28.1%) patients developed tumor progression and 58 (12.7%) patients died. In the validation cohort, there were 179 (77.2%) male and 53 (22.8%) female patients with a median age of 47 years. The median follow-up time was 61.5 months. During follow-up, 74 (31.9%) and 35 (15.1%) patients experienced tumor progression and mortality, respectively.

### The optimal cutoff of LA and EBV DNA

**Fig. 1:**
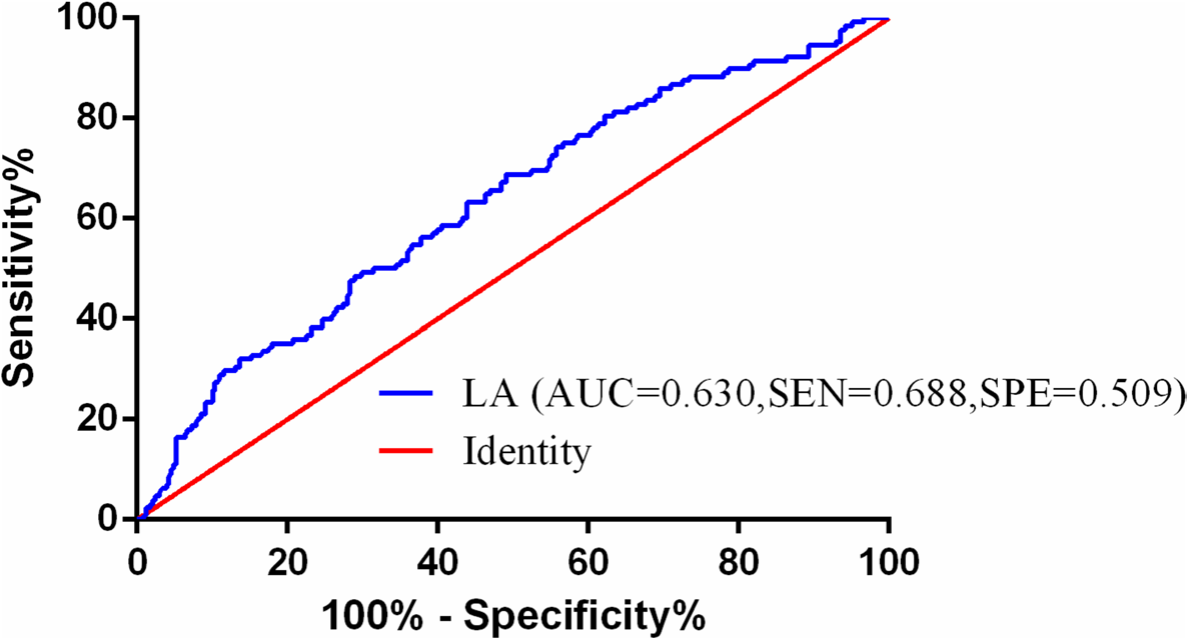
Receiver operating characteristic curve for pretreatment LA based on PFS. The area of LA under the curve is 0.630, 95% CI: 0.573–0.687, *P* < 0.001; sensitivity: 0.688, specificity: 0.509

The optimal cutoff value for LA was 80.17, according to the ROC curve (AUC: 0.630, 95% CI: 0.573–0.687, *P* < 0.001; sensitivity: 0.688, specificity: 0.509) (Fig. 1). Based on the cutoff above, the patients were divided into low and high LA groups. Regarding EBV DNA, the referring threshold in our institution was 500 copies/mL. Therefore, we chose this value as the optimal cut-off value to classify patients into negative and positive groups.

### Patient characteristics according to LA in the two cohorts

Table 2 presents the association between LA and patient clinical characteristics. The results revealed that LA was only correlated with sex in both the training (*P* = 0.046) and validation (*P* = 0.035) cohorts. However, in the validation cohort, a significant correlation between LA and age (*P* = 0.006) and EBV DNA (*P* = 0.036) was also observed.

**Table 2:** Baseline characteristics of the patients in training and validation cohorts according to LA

Characteristic	Training cohort			Validation cohort		
	LA (≤ 80.17)	LA (> 80.17)	*P* value	LA (≤ 80.17)	LA (> 80.17)	*P* value
	(*n* = 249)	(*n* = 207)		(*n* = 115)	(*n* = 117)	
Gender (male/female)	169/80	158/49	**0.046**	82/33	97/20	**0.035**
Age (≤ 55/ > 55,years)	189/60	163/44	0.472	85/30	103/14	**0.006**
Smoke (no/yes)	155/94	123/84	0.538	69/46	61/56	0.228
Drink (no/yes)	224/25	191/16	0.390	101/14	101/16	0.733
WHO pathological type (I/II/III)	0/18/231	0/14/193	1.000	1/6/108	2/8/107	0.837
EBV DNA (negative/positive)	125/124	86/121	0.065	59/56	44/73	**0.036**
T stage (T1-T2/T3-T4)	93/156	86/121	0.361	46/69	58/59	0.143
N stage (N0-N1/N2-N3)	111/138	91/116	0.895	49/66	56/61	0.421
TNM stage (I-II/III-IVa)	46/203	49/158	0.174	20/95	32/85	0.069

*Abbreviations: LA* Lymphocyte × albumin, *WHO* World Health Organization, *EBV DNA* Epstein-Barr virus DNA, *T* Tumor, *N* Node, *TNM* Tumor node metastasis

### Survival curves of LA and EBV DNA

**Fig. 2:**
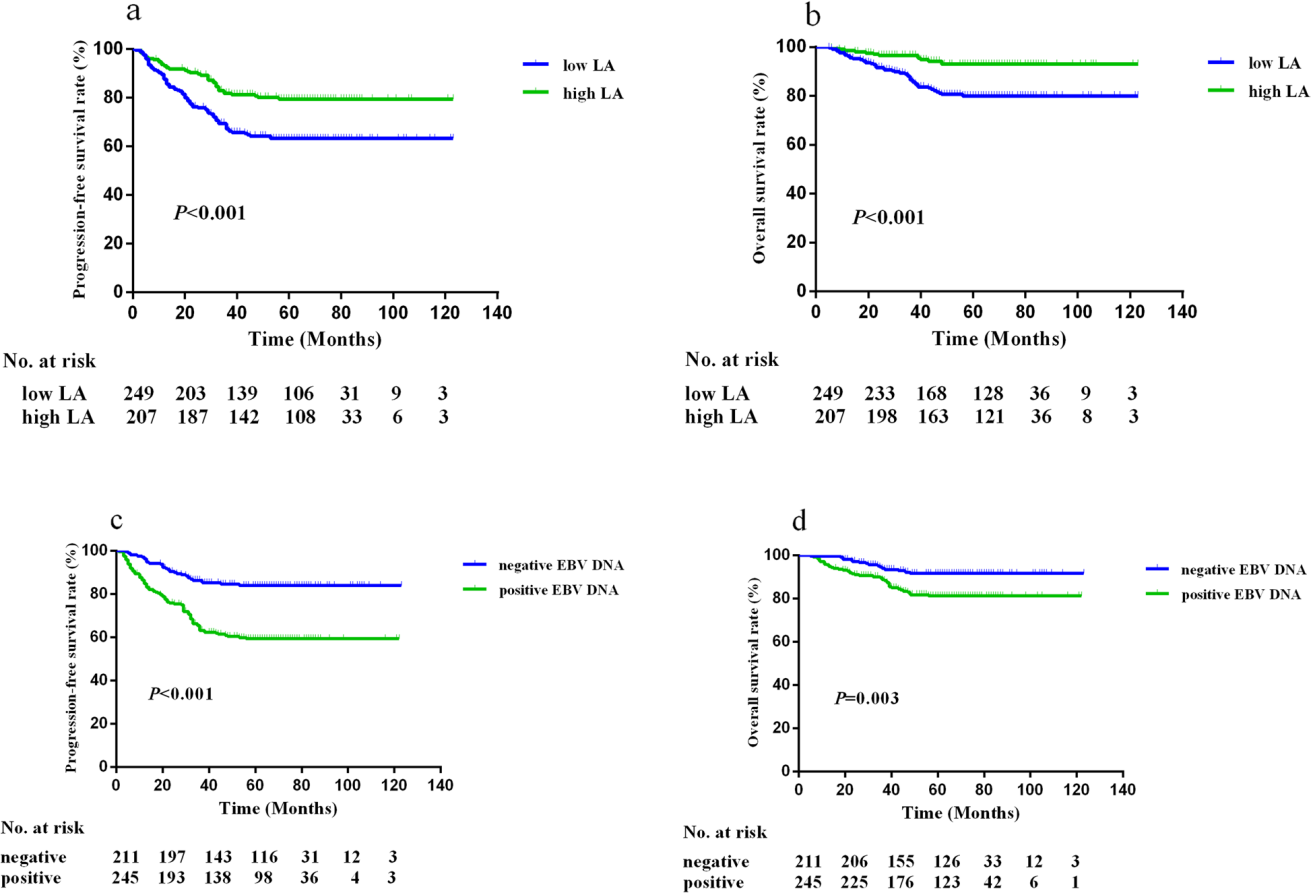
Kaplan–Meier survival curves of PFS and OS in the training cohort. **a** shows the curves based on LA groups for PFS; **b** shows the curves based on LA groups for OS; **c** shows the curves based on EBV DNA groups for PFS; **d** shows the curves based on EBV DNA groups for OS. Worse prognosis was observed in patients with low LA or positive EBV DNA

In the training cohort, the Kaplan–Meier survival analyses revealed that compared with the higher LA group, the low LA group had shorter PFS and OS (all *P* < 0.001, Fig. 2a, b). In the level of EBV DNA, patients with positive EBV DNA had worse PFS (*P* < 0.001, Fig. 2c) and OS (*P* = 0.003, Fig. 2d) than patients with negative EBV DNA. The above results were confirmed in the validation cohort (all *P* < 0.01, See Supplementary Figure 1, Additional File 3).

Besides, we further performed subgroup analyses to assess the prognostic value of LA and EBV DNA in the patients groups like gender, age and TNM stage. Only in the TNM stage groups we found the difference between LA or EBV DNA groups. It showed that in the TNM stage subgroup of III-IVa, patients with low LA levels or positive EBV DNA had poor PFS (*P* < 0.001) and OS (*P* < 0.010). The results were also observed in validation cohorts. (See Supplementary Figures 2–3, Additional Files 4 and 5).

### Univariate and multivariate analyses

In univariate analyses, the variables of age, T stage, TNM stage, LA, and EBV DNA were significant predictors of PFS and OS. Gender was also a significant predictor of PFS (*P* = 0.042). All variables with *P* values less than 0.05 in univariate analysis were included in multivariate Cox analysis.

Multivariate analyses demonstrated that LA, EBV DNA, and TNM stage were significant independent prognostic factors associated with OS and PFS (all *P* < 0.050). In addition, age > 55 years was still found to be an independent risk factor for OS (*P* < 0.001). The complete results are presented in Tables 3 and 4.

**Table 3:** Univariate Cox analyses of PFS and OS in training and validation cohorts

Characteristics	Training cohort		Validation cohort	
	HR (95% CI)	*P* value	HR (95% CI)	*P* value
**PFS**				
Gender (Male vs. Female)	0.644(0.421–0.985)	**0.042**	1.017(0.591–1.747)	0.952
Age (≤ 55 vs. > 55)	1.754(1.209–2.543)	**0.003**	1.595(0.938–2.713)	0.085
Smoke (no vs. yes)	1.177(0.829–1.672)	0.362	1.003(0.633–1.588)	0.991
Drink (no vs. yes)	0.883(0.463–1.685)	0.707	0.934(0.465–1.876)	0.848
T stage (T1-T2 vs. T3-T4)	1.825(1.241–2.683)	**0.002**	1.432(0.897–2.284)	0.132
N stage (N0-N1 vs. N2-N3)	1.347(0.945–1.919)	0.099	2.102(1.284–3.440)	**0.003**
TNM stage (I-II vs. III-IVa)	3.355(1.808–6.228)	** < 0.001**	2.456(1.223–4.934)	**0.012**
EBV DNA (negative vs. positive)	2.966(1.987–4.425)	** < 0.001**	2.992(1.758–5.093)	** < 0.001**
LA (≤ 80.17 vs. > 80.17)	0.495(0.340–0.719)	** < 0.001**	0.463(0.287–0.747)	**0.002**
**OS**				
Gender (Male vs. Female)	0.802(0.440–1.464)	0.472	0.712(0.296–1.716)	0.449
Age (≤ 55 vs. > 55)	3.595(2.146–6.021)	** < 0.001**	2.910(1.465–5.779)	**0.002**
Smoke (no vs. yes)	1.206(0.717–2.027)	0.481	1.366(0.704–2.652)	0.356
Drink (no vs. yes)	1.200(0.516–2.795)	0.672	1.688(0.737–3.864)	0.216
T stage (T1-T2 vs. T3-T4)	2.007(1.115–3.614)	**0.020**	2.583(1.210–5.512)	**0.014**
N stage (N0-N1 vs. N2-N3)	1.313(0.776–2.222)	0.311	1.655(0.823–3.326)	0.157
TNM stage (I-II vs. III-IVa)	8.462(2.065–34.683)	**0.003**	5.283(1.268–22.022)	**0.022**
EBV DNA (negative vs. positive)	2.358(1.326–4.194)	**0.004**	2.914(1.324–6.416)	**0.008**
LA (≤ 80.17 vs. > 80.17)	0.324(0.175–0.601)	** < 0.001**	0.219(0.096–0.502)	** < 0.001**

*Abbreviations: T* Tumor, *N* Node, *TNM* Tumor node metastasis, *EBV DNA* Epstein-Barr virus DNA, *HR* Hazard ratio, *CI* Confidence interval, *LA* Lymphocyte × albumin, *PFS* Progression-free survival, *OS* Overall survival

**Table 4:** Multivariate Cox analyses of PFS and OS in training and validation cohorts

Characteristics	Training cohort		Validation cohort	
	HR (95% CI)	*P* value	HR (95% CI)	*P* value
**PFS**				
Gender (Male vs. Female)	0.704(0.458–1.082)	0.110	-	-
Age (≤ 55 vs. > 55)	1.424(0.973–2.084)	0.069	-	-
T stage (T1-T2 vs. T3-T4)	0.962(0.615–1.506)	0.866	-	-
N stage (N0-N1 vs. N2-N3)	-	-	1.310(0.720–2.385)	0.376
TNM stage (I-II vs. III-IVa)	2.680(1.314–5.466)	**0.007**	1.536(0.664–3.555)	0.316
EBV DNA (negative vs. positive)	2.625(1.737–3.966)	** < 0.001**	3.237(1.870–5.604)	** < 0.001**
LA (≤ 80.17 vs. > 80.17)	0.463(0.318–0.675)	** < 0.001**	0.392(0.241–0.637)	** < 0.001**
**OS**				
Age (≤ 55 vs. > 55)	3.163(1.860–5.380)	** < 0.001**	2.488(1.244–4.974)	**0.010**
T stage (T1-T2 vs. T3-T4)	0.788(0.419–1.481)	0.459	1.815(0.783–4.206)	0.165
TNM stage (I-II vs. III-IVa)	7.890(1.770–35.170)	**0.007**	2.698(0.558–13.035)	0.217
EBV DNA (negative vs. positive)	1.950(1.083–3.510)	**0.026**	3.703(1.659–8.263)	**0.001**
LA (≤ 80.17 vs. > 80.17)	0.340(0.182–0.633)	**0.001**	0.206(0.089–0.479)	** < 0.001**

*Abbreviations: T* Tumor, *N* Node, *TNM* Tumor node metastasis, *EBV DNA* Epstein-Barr virus DNA, *HR* Hazard ratio, *CI* Confidence interval, *LA* Lymphocyte × albumin, *PFS* Progression-free survival, *OS* Overall survival

### Prognostic value of EBV DNA combined with LA

**Fig. 3:**
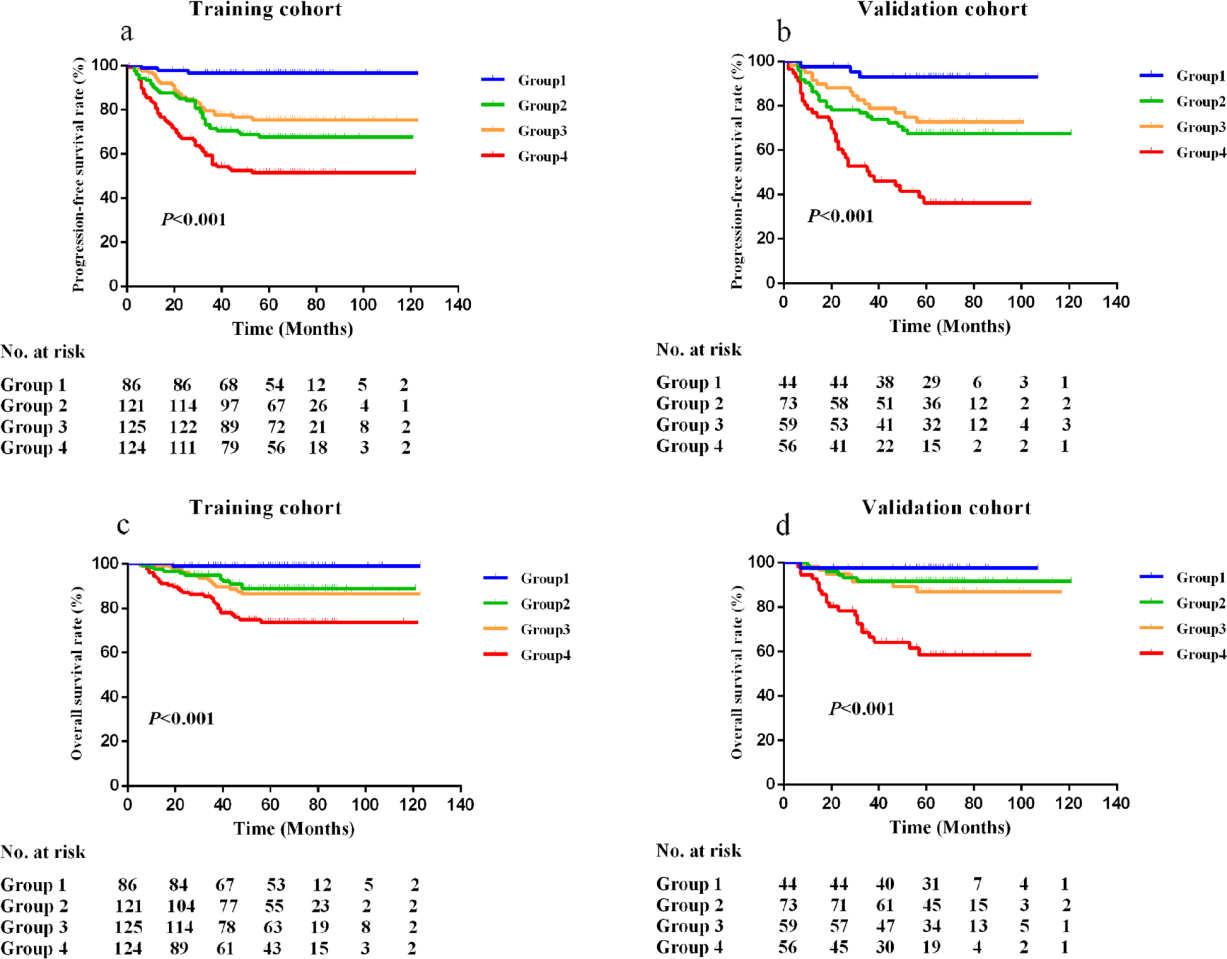
Kaplan–Meier survival curves of PFS and OS based on different groups between LA combined with EBV DNA in the training and validation cohorts. **a** Survival curves for PFS in the training cohort; **b** Survival curves for PFS in the validation cohort; **c** Survival curves for OS in the training cohort; **d** Survival curves for OS in the validation cohort. Worse prognosis was observed in patients with Group 4 (patients with low LA and positive EBV DNA)

Since LA and EBV DNA were both independent prognostic factors in the multivariate analysis, we further combined LA and EBV DNA to explore its prognostic value. Patients were distributed into four groups: Group 1, high LA + negative EBV DNA; Group 2, high LA + positive EBV DNA; Group 3, low LA + negative EBV DNA; and Group 4, low LA + positive EBV DNA. In the training cohort, 86 (18.9%), 121 (26.5%), 125 (27.4%), and 124 (27.2%) patients were assigned to each group. Our results revealed a significant survival difference in LA combined with EBV DNA. Patients in Group 4 had obviously worse PFS and OS than patients in the other groups (all *P* < 0.001, Fig. 3a, c). The results were verified in the validation cohort (Fig. 3b, d).

### Nomograms establishment and validation

**Fig. 4:**
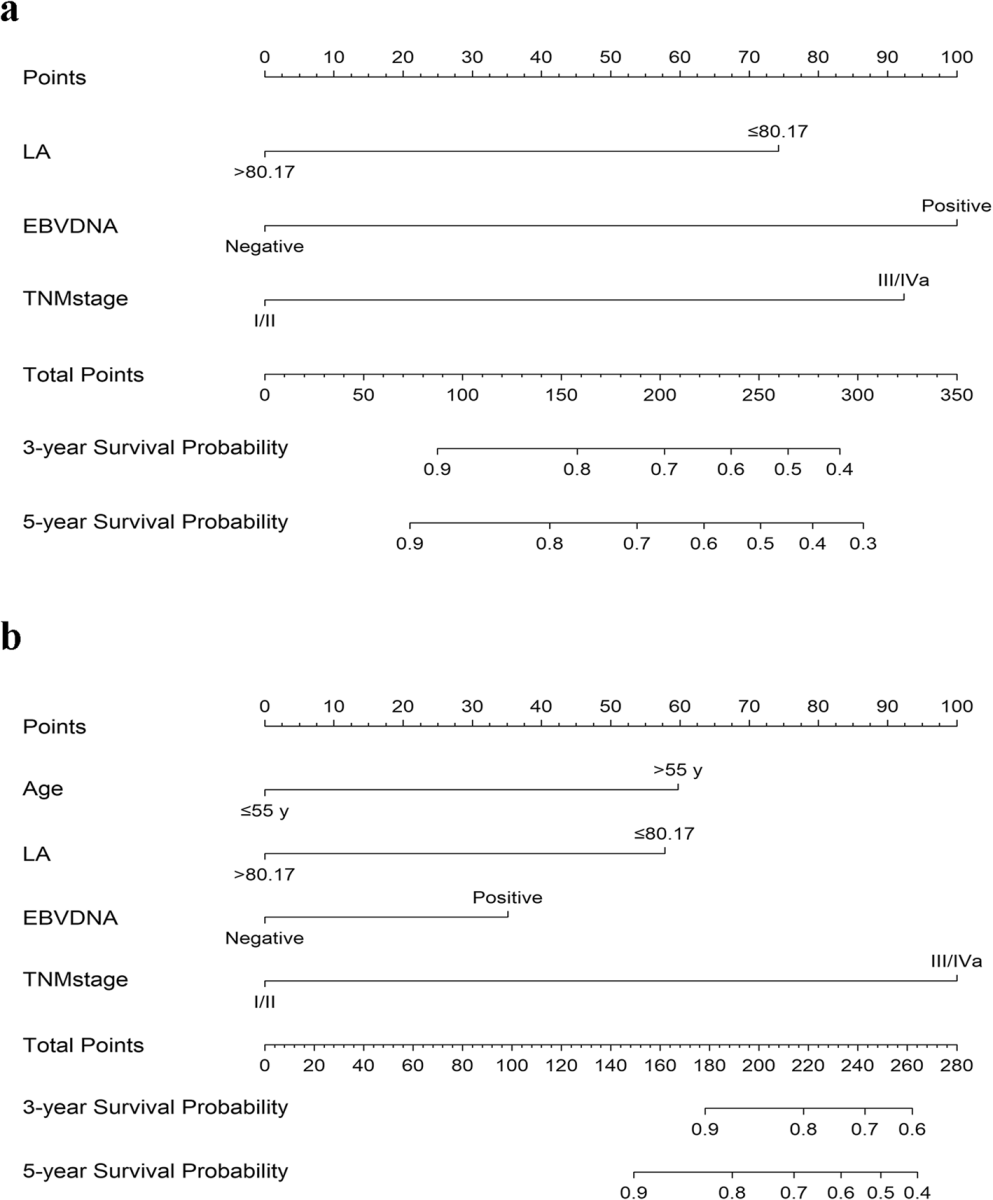
Nomogram predicting 3- and 5-year progression-free survival (**a**) and overall survival (**b**) in NPC patients. A vertical line is drawn from each factor, each factor was assigned to a point score, and the corresponding points represent how much the factor contributed to the risk. Summing these points to generate a total score by drawing a vertical line to the bottommost line, it can translate into the 3- and 5-year PFS or OS probabilities

To enhance the accuracy of individual PFS and OS predictions, we constructed nomograms of PFS and OS based on the results of multivariate analyses in the training cohort. According to the multivariate analyses, three independent prognostic variables (TNM stage, LA, and EBV DNA) were integrated into the PFS nomogram (Fig. 4a), while the OS nomogram was constructed using the four independent prognostic factors: age, TNM stage, LA, and EBV DNA (Fig. 4b). Each variable was assigned a point within the nomograms. By calculating these total points and placing them on the score scale we can estimate the individual probabilities of 3- and 5-year PFS or OS.

**Fig. 5:**
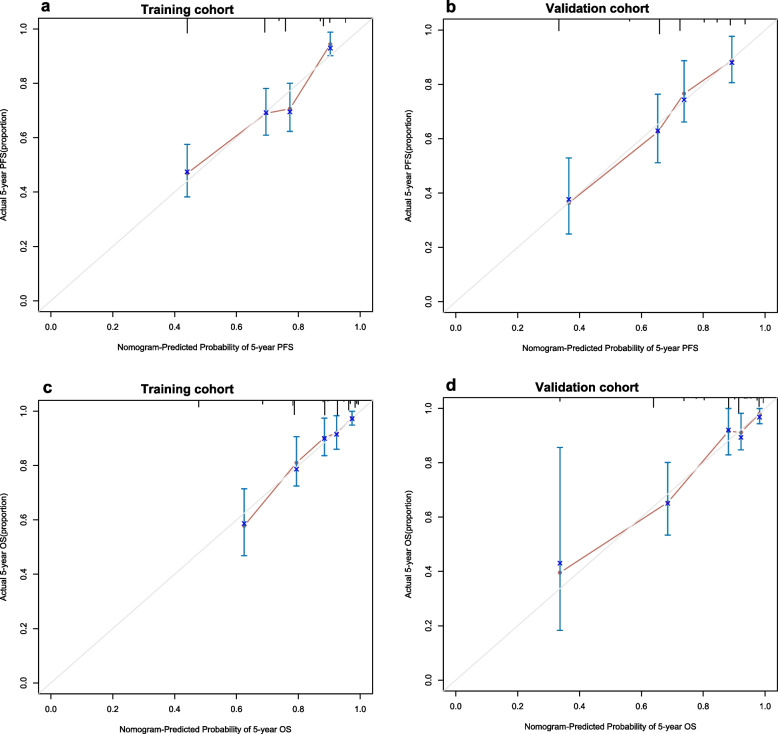
The calibration curves for predicting the 5-year PFS and OS. **a** Prediction of PFS in the training cohort; **b** Prediction of PFS in the validation cohort; **c** Prediction of OS in the training cohort; **d** Prediction of OS in the validation cohort. The red line represents the nomogram’s performance. Red dots with blue bars represent the nomogram’s performance with 95% CI when applied to the observed surviving cohorts. The closer the nomogram curve is to the diagonal line, the more closely the predicted probability matches the actual probability

**Fig. 6:**
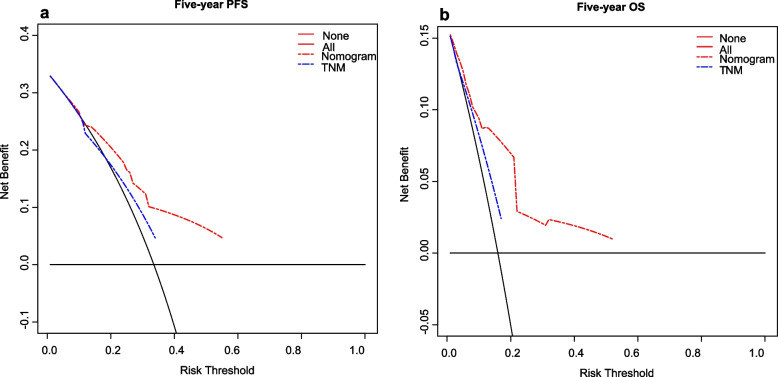
The decision curve analysis for predicting the 5-year PFS (**a**) and OS (**b**). The x-axis was determined by the threshold probability. The y-axis was a net benefit, which was the relative benefit derived from the proportion of true-positive results subtracted from the proportion of false-positive results weighted by a ratio of threshold probabilities. Under the same probability, the clinical usefulness was better when the net benefit was higher. The blue line represents the clinical net benefit of TNM, and the red line represents the clinical net benefit of our nomogram. Our nomogram had more clinical net benefit than TNM stage

The calibration plots showed great consistency between actual observation and the nomogram prediction of 3-year and 5- year PFS and OS (See in Supplementary Figure 4, Additional File 6 and Fig. 5), whether in the training cohort or in the validation cohort. The nomogram for predicting PFS had a C-index of 0.69 (95% CI: 0.65–0.73) in the training cohort and 0.70 (95% CI:0.65–0.75) in the validation cohort (Table 5). For the OS nomogram, the values were 0.77 (95% CI: 0.71–0.82) and 0.77 (95% CI:0.70–0.85), respectively. The C-index was higher than those of TNM stage (PFS: 0.58; OS: 0.60), EBV DNA (PFS: 0.63; OS: 0.60), and LA (PFS: 0.59; OS: 0.62). Both PFS and OS nomograms showed satisfactory model performance. Furthermore, DCA curve analyses demonstrated that the nomograms based on LA and EBV DNA achieved higher net clinical benefits in predicting PFS and OS (Fig. 6).

**Table 5:** Comparison of the C-index of different models

Variables	Training cohort		Validation cohort	
	C-index	95% CI	C-index	95% CI
PFS				
Nomogram	0.69	0.65–0.73	0.70	0.65–0.75
TNM stage	0.58	0.55–0.61	0.57	0.53–0.61
TNM stage + LA	0.64	0.60–0.68	0.62	0.56–0.68
TNM stage + EBV	0.65	0.62–0.69	0.65	0.60–0.71
LA + EBV	0.67	0.63–0.72	0.68	0.63–0.74
LA	0.59	0.54–0.63	0.59	0.53–0.65
EBV DNA	0.63	0.59–0.66	0.63	0.58–0.68
OS				
Nomogram	0.77	0.71–0.82	0.77	0.70–0.85
TNM stage	0.60	0.57–0.63	0.59	0.55–0.64
TNM stage + LA	0.69	0.64–0.74	0.69	0.62–0.77
TNM stage + EBV	0.66	0.61–0.72	0.67	0.60–0.74
LA + EBV	0.68	0.62–0.74	0.73	0.65–0.81
LA	0.62	0.57–0.68	0.66	0.59–0.73
EBV DNA	0.60	0.54–0.66	0.62	0.55–0.69

*Abbreviations:* *C-index* Concordance index, *CI* Confidence interval, *TNM* Tumor node metastasis, *LA* Lymphocyte × albumin, *EBV DNA* Epstein-Barr virus DNA, *PFS* Progression-free survival, *OS* Overall survival

## Discussion

Owing to tumor heterogeneity, personalized treatment has become an important developing strategy for cancer treatment. Precise prognosis estimation helps clinicians tailor treatment options to improve survival outcomes. Hence, further refined risk stratification of patients with cancer is indispensable for individual treatment. Our study confirmed that LA was a strong independent factor for predicting clinical outcomes. The combination of LA and EBV DNA provided more detailed information for patient stratification. Moreover, we constructed comprehensive prognostic nomograms incorporating LA and EBV DNA for PFS and OS prediction. The C-index, calibration curve, and DCA curve demonstrated that this model achieved excellent predictive efficiency and clinical benefits.

To the best of our knowledge, this is the first study to focus on the value of LA in NPC. We noticed that a low LA was obviously related to poorer 5-year PFS and OS in both the training and validation cohorts. This suggests that patients with low lymphocyte and low albumin levels had worse survival outcomes, which is consistent with previous studies [16, 31–33]. Multivariate analysis also showed that low LA levels were an independent risk factor for NPC. Although the underlying mechanism between LA and poor cancer prognosis is unclear, a possible explanation can be proposed.

The interactions between immune environment and cancer cells are dynamic and complex, and the immune biomarkers can reveal the prognosis of cancer patients [7]. Lymphocytes are one of the critical cells in the immune response. When tumor growth, its tumor antigens can be recognized by lymphocytes, then the secretion of cytokines activate different kinds of immune cells to exhibit cancer inhibitory effects such as CD4 + and CD8 + T cells, B cells and so on [34–37]. In this regard, a low peripheral lymphocyte level may reflect a poor lymphocyte-mediated antitumor immune reaction and worse immune surveillance, indicate a poor prognosis. Previous study also showed that circulating lymphocytes can improve cancer patient prognosis by enhancing cancer immune regulation and inhibiting cancer cell proliferation [38].

Moreover, proinflammatory cytokines can not only promote tumor invasion but also inhibit the synthesis of albumin and lead to its leakage by increasing microvascular permeability [10, 39, 40]. Thus, as the disease progresses, albumin levels decrease significantly and lead to malnutrition. Since the key roles of nutrition in determining the fate and functions of immune cells, poor nutritional status could induce an impaired immune response, which promotes cancer progression and causes a worse survival outcomes [41]. Given these findings, a low LA level, multiplied by a low lymphocyte count and low albumin level, might reflect the status of tumor progression, poor immune response and malnutrition at the same time, with a higher predictive accuracy. Therefore, patients with low LA require more aggressive treatment regimens.

Unlike LA, EBV DNA has been recognized as an important indicator of the prognosis of NPC. Studies indicated that EBV DNA was originated from NPC tumor cells, is a fragment of tumor cells necrosis and lysis, and its plasma load can reflect tumor load [42–44]. Similar to our results, the subgroups analyses of TNM stage revealed that positive EBV DNA was obviously with worse PFS and OS in the III-IVa TNM stage patients. To strengthen the predictive value, Xiong [29] et al. combined systemic immune-inflammation index (SII) and EBV DNA. The study revealed that patients with a high SII and positive EBV DNA had a higher risk of death and disease progression. These results are in line with our research. We combined EBV DNA and LA, and found that the C-index of EBV DNA in combination with LA for PFS and OS was 0.67 and 0.68, respectively, which was higher than that of EBV DNA (PFS: 0.63, OS: 0.60), and even higher than the lactate dehydrogenase/ albumin ratio (LAR) in the research by Zhu et al. [30] and SII in the study by Xiong et al. [29]. Moreover, Kaplan–Meier curves showed that compared with patients with high LA and negative EBV DNA, patients with low LA and positive EBV DNA were associated with a higher risk of tumor progression. This suggests that EBV DNA combined with LA has a superior prognostic value than EBV DNA alone. This integrated biomarker indeed improves the accuracy of outcome prediction. The above results strongly indicate that EBV DNA combined with LA is more effective for further risk stratification in NPC patients. They should be given much more attention, and more chemotherapy cycle, nutrition improvement, targeted therapy, and even immunotherapy need to be considered early to improve the clinical outcomes. Then, we developed nomograms based on LA and EBV DNA and other clinical characteristics.

Recently, nomograms, with their simple, intuitive, and individual characteristics, have been widely used in cancer prognosis prediction. In studies conducted by Tang et al. [45] and Zhang et al. [46], nomograms were a convenient and reliable tool to estimate the survival of NPC patients, and they provided excellent discrimination capacity compared to the current TNM staging system. Consistent with our results, we found that the C-indexes (PFS: 0.69, OS: 0.77) of the nomograms outperformed the TNM staging system (PFS: 0.58, OS: 0.6), which meant that the nomograms were superior in risk stratification. Furthermore, the DCA analysis results further supported that it was significantly better than the TNM staging system in clinical application. Through nomograms, clinicians could more accurately and efficiently identify patients with a high risk of poor clinical outcomes and make the personalized treatment plans. For high-risk patients, more aggressive therapy may be considered to improve their survival.

However, there are some limitations to our study. First, this was a retrospective study. Although we conducted an internal validation to make the results more convincing, it is still far from practical clinical application. Second, in addition to LA, many other indicators such as LDH, CAR, SII and SIRI were reportedly associated with the prognosis of NPC patients. On account of the limited sample size, these relations have not been fully explored. Third, it was a single-center study with the absence of external validation. Hence, a large-scale and multi-center prospective study is required to further validate the value of LA.

## Conclusions

In conclusion, our study reported that LA was a simple and easily available biomarker, and when combined with EBV DNA, it was a stronger predictor of PFS and OS in NPC. The nomogram based on LA and EBV DNA is more effective and precise in predicting individual survival outcomes for NPC patients. It can serve as a good supplement to the TNM staging system to improve the identification of high risk of disease progression in NPC patients and guide more aggressive treatments for these patients to prolong their survival.

### Supplementary Information

**Additional file 1: Supplementary file 1.** The method of EBV DNA detection.

**Additional file 2: Supplementary file 2.** The detailed protocols for radiotherapy and chemotherapy.

**Additional file 3: Supplementary Figure 1.** Kaplan–Meier survival curves of PFS and OS in the validation cohort.

**Additional file 4: Supplementary Figure 2.** Kaplan–Meier survival curves of PFS and OS in the subgroup analyses of TNM stage in the training cohort.

**Additional file 5: Supplementary Figure 3.** Kaplan–Meier survival curves of PFS and OS in the subgroup analyses of TNM stage in the validation cohort.

**Additional file 6: Supplementary Figure 4.** The calibration curves for predicting the 3-year PFS and OS.

## Data Availability

The data used to support the findings of this study are available from the corresponding author on reasonable request.

## Abbreviations

ALB: Albumin
AJCC: American Joint Committee On Cancer
AUC: Area Under The Curve
AC: Adjuvant Chemotherapy
C-index: Concordance index
CAR: C-reactive protein/ Albumin Ratio
CI: Confidence Interval
CCRT: Concurrent Chemoradiotherapy
DCA: Decision Curve Analysis
EBV: Epstein–Barr Virus
HR: Hazard Ratio
IMRT: Intensity-Modulated Radiation Therapy
IC: Induction Chemotherapy
IL-2: Interleukin-2
IFN-γ: Interferon-gamma
LA: Lymphocytes × Albumin
LAR: Lactate dehydrogenase/ Albumin Ratio
LDH: Lactate Dehydrogenase
MRI: Magnetic Resonance Imaging
NPC: Nasopharyngeal Carcinoma
NCCN: National Comprehensive Cancer Network
OS: Overall Survival
PET-CT: Positron Emission Tomography-Computed Tomography
PFS: Progression-free Survival
ROC: Receiver Operating Characteristic
SII: Systemic Immune-inflammation Index
SIRI: Systemic Inflammation Response Index
TILs: Tumor-Infiltrating Lymphocytes
TNM: Tumor Node Metastasis
