# Supplementary material for: Nomogram incorporating Epstein-Barr virus DNA and a novel immune-nutritional marker for survival prediction in nasopharyngeal carcinoma
Source: BMC Cancer. 2023 Dec 9;23:1217. doi: 10.1186/s12885-023-11691-8 (PMC10709872; doi:10.1186/s12885-023-11691-8)
Supplement: Supplementary file 1 — Additional file 1: Supplementary file 1. The method of EBV DNA detection. [file 12885_2023_11691_MOESM1_ESM.docx]

**Supplementary file 1:**

**The methods of EBV DNA detection**

Plasma EBV DNA measurements were completed in the Laboratory Medicine Center of Nanfang Hospital, Southern Medical University. Before treatment, venous blood samples (5ml/each case) were collected into ehylenediaminetetraacetic acid (EDTA) tubes, then centrifuged at 1500×g for 5 min at 4°C. Plasma total DNA was extracted by the QIAamp blood kit (Qiagen, Hilden, Germany). The BamH I-W region of EBV genome was amplified by real-time quantitative polymerase-chain reaction (RT-qPCR) using an EBV RT-qPCR kit and primers 5′-GCTGCGCTGCTGCTATCTT-3′ (forward) and 5′-CAAGCCCACTCCCCTGTCT-3′ (reverse) according to the manufacturer’s instructions (Liferiver, Shanghai, China). The GAPDH gene was amplified as a control using the primers 5′-GGCGACGCAAAAGAAGATG-3′ (forward) and 5′-CCGTTGACTCCGACCTTCAC-3′ (reverse). PCR conditions were as follows: initial denaturation at 95°C for 10 minutes, followed by 40 cycles of denaturation at 95°C for 15 seconds and amplification at 56°C for 1 minute.
