# Supplementary material for: Nomogram incorporating Epstein-Barr virus DNA and a novel immune-nutritional marker for survival prediction in nasopharyngeal carcinoma
Source: BMC Cancer. 2023 Dec 9;23:1217. doi: 10.1186/s12885-023-11691-8 (PMC10709872; doi:10.1186/s12885-023-11691-8)
Supplement: Supplementary file 2 — Additional file 2: Supplementary file 2. The detailed protocols for radiotherapy and chemotherapy. [file 12885_2023_11691_MOESM2_ESM.docx]

**Supplementary file 2:**

**The details of radiotherapy and chemotherapy**

All patients received IMRT with the daily fraction raging from 2.12-2.24 Gy, 5 days a week for 6-8 weeks. The total prescribed doses were 70-74 Gy to the primary tumor volume of the nasopharynx (GTVnx), 66-70 Gy to the the positive neck lymph node tumor volume (GTVnd), 60-62 Gy to the high-risk clinical target volume 1 (CTV1)，and 50-56 Gy to the low-risk clinical target volume 2 (CTV2).

Concurrent chemotherapy (1-2 cycles) included cisplatin monotherapy regimen (cisplatin 25 mg/m^2^/d on days 1-3) and TP regimen (paclitaxel 135 mg/m^2^/d or docetaxel 60 mg/m^2^/d,day 1; cisplatin 25 mg/m^2^/d on days 1-3). Induction chemotherapy (1-2 cycles) or adjuvant chemotherapy (1-4 cycles) was performed with TP (paclitaxel 135 mg/m^2^/d or docetaxel 60 mg/m^2^/d,day 1; cisplatin 25 mg/m^2^/d on days 1-3) or TPF (paclitaxel 135 mg/m^2^/d or docetaxel 60 mg/m^2^/d,day 1; cisplatin 25 mg/m^2^/d on days 1-3; 5-fluorouracil 600 mg/m^2^/d on days 1-5), or PF (cisplatin 25 mg/m^2^/d on days 1-3; 5-fluorouracil 600 mg/m^2^/d on days 1-5). All of the chemotherapy regimens were performed in every 21 days as a cycle.
