# Supplementary material for: Nomogram incorporating Epstein-Barr virus DNA and a novel immune-nutritional marker for survival prediction in nasopharyngeal carcinoma
Source: BMC Cancer. 2023 Dec 9;23:1217. doi: 10.1186/s12885-023-11691-8 (PMC10709872; doi:10.1186/s12885-023-11691-8)
Supplement: Supplementary file 3 — Additional file 3: Supplementary Figure 1. Kaplan–Meier survival curves of PFS and OS in the validation cohort. [file 12885_2023_11691_MOESM3_ESM.docx]

**Supplementary Figure 1:**

**

**

**Fig. S1** Kaplan–Meier survival curves of PFS and OS in the validation cohort. (a) shows the curves based on LA groups for PFS; (b) shows the curves based on LA groups for OS; (c) shows the curves based on EBV DNA groups for PFS; (d) shows the curves based on EBV DNA groups for OS. Worse prognosis was observed in patients with low LA or positive EBV DNA
