# Supplementary material for: Nomogram incorporating Epstein-Barr virus DNA and a novel immune-nutritional marker for survival prediction in nasopharyngeal carcinoma
Source: BMC Cancer. 2023 Dec 9;23:1217. doi: 10.1186/s12885-023-11691-8 (PMC10709872; doi:10.1186/s12885-023-11691-8)
Supplement: Supplementary file 4 — Additional file 4: Supplementary Figure 2. Kaplan–Meier survival curves of PFS and OS in the subgroup analyses of TNM stage in the training cohort. [file 12885_2023_11691_MOESM4_ESM.docx]

**Supplementary Figure 2:**





**Fig. S2** Kaplan–Meier survival curves of PFS and OS in the subgroup analysis of TNM stage in the training cohort. (a-d) shows the curves based on LA groups for PFS and OS in the subgroup analysis of TNM stage; (e-h) shows the curves based on EBV DNA groups for PFS and OS in the subgroup analysis of TNM stage. In the TNM stage subgroup of Ⅲ-Ⅳa, patients with low LA levels or positive EBV DNA had poor PFS
