# Supplementary material for: Nomogram incorporating Epstein-Barr virus DNA and a novel immune-nutritional marker for survival prediction in nasopharyngeal carcinoma
Source: BMC Cancer. 2023 Dec 9;23:1217. doi: 10.1186/s12885-023-11691-8 (PMC10709872; doi:10.1186/s12885-023-11691-8)
Supplement: Supplementary file 6 — Additional file 6: Supplementary Figure 4. The calibration curves for predicting the 3-year PFS and OS. [file 12885_2023_11691_MOESM6_ESM.docx]

**Supplementary Figure 4:**

**
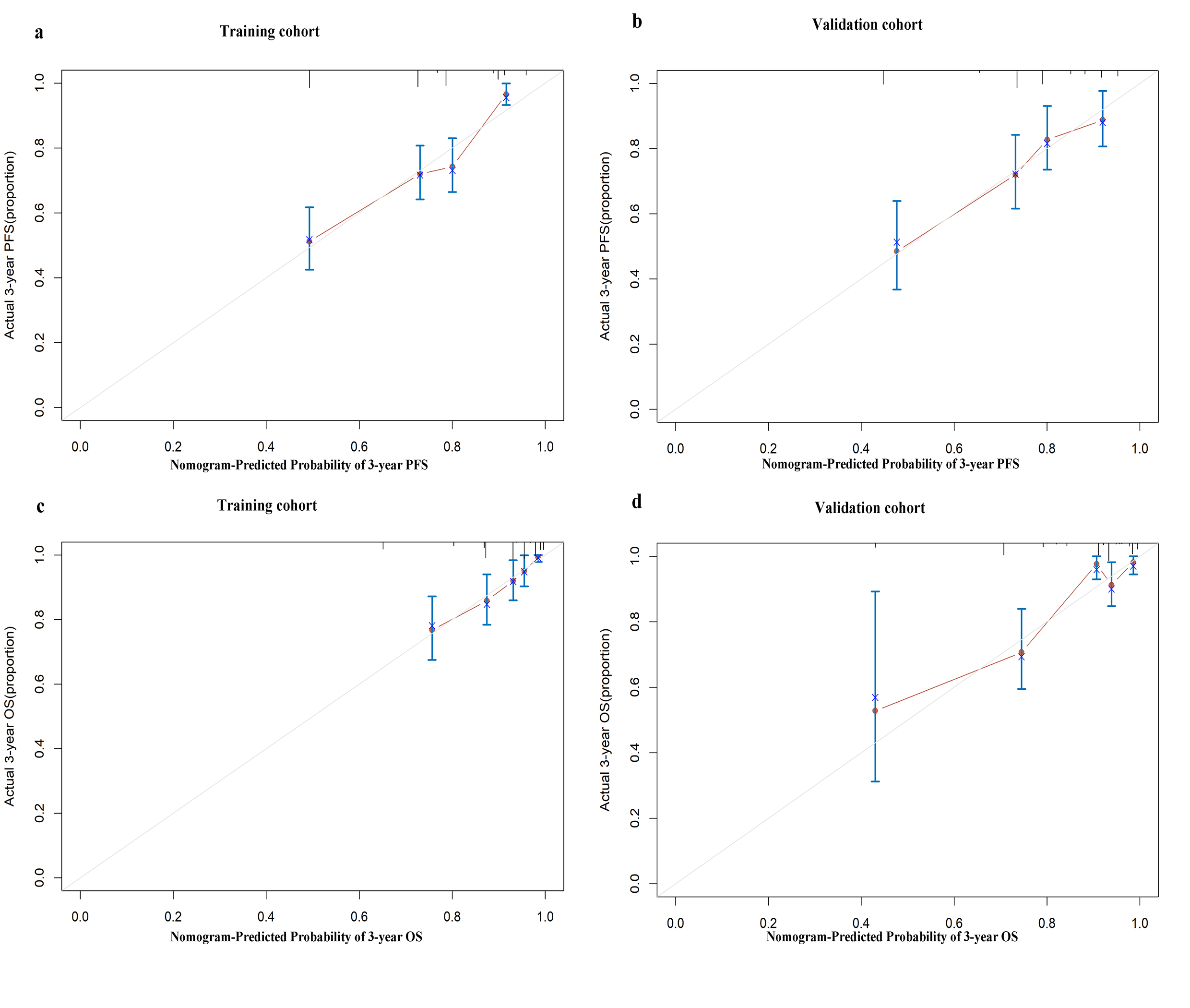
**

**Fig. S4** The calibration curves for predicting the 3-year PFS and OS. (a) Prediction of PFS in the training cohort; (b) Prediction of PFS in the validation cohort; (c) Prediction of OS in the training cohort; (d) Prediction of OS in the validation cohort. The red line represents the nomogram’s performance. Red dots with blue bars represent the nomogram’s performance with 95% CI when applied to the observed surviving cohorts. The closer the nomogram curve is to the diagonal line, the more closely the predicted probability matches the actual probability
